# Cellular and Humoral Immune Response to a Third Dose of BNT162b2 COVID-19 Vaccine – A Prospective Observational Study

**DOI:** 10.3389/fimmu.2022.896151

**Published:** 2022-07-01

**Authors:** Jonas Herzberg, Bastian Fischer, Heiko Becher, Ann-Kristin Becker, Human Honarpisheh, Salman Yousuf Guraya, Tim Strate, Cornelius Knabbe

**Affiliations:** ^1^ Department of Surgery – Krankenhaus Reinbek St. Adolf-Stift, Reinbek, Germany; ^2^ Institut für Laboratoriums- und Transfusionsmedizin, Herz- und Diabeteszentrum NRW, Bad Oeynhausen, Germany; ^3^ Institute of Medical Biometry and Epidemiology, University Medical Center Hamburg-Eppendorf, Hamburg, Germany; ^4^ Asklepios Klinik Harburg, Abteilung für Psychiatrie und Psychotherapie, Hamburg, Germany; ^5^ Clinical Sciences Department, College of Medicine, University of Sharjah, Sharjah, United Arab Emirates

**Keywords:** SARS-CoV-2, humoral and cellular immunity, seroprevalence, vaccination, BNT162b2, third dose

## Abstract

**Background:**

Since the introduction of various vaccines against SARS-CoV-2 at the end of 2020, infection rates have continued to climb worldwide. This led to the establishment of a third dose vaccination in several countries, known as a booster. To date, there has been little real-world data about the immunological effect of this strategy.

**Methods:**

We compared the humoral- and cellular immune response before and after the third dose of BioNTech/Pfizer vaccine BNT162b2, following different prime-boost regimen in a prospective observational study. Humoral immunity was assessed by determining anti-SARS-CoV-2 binding antibodies using a standardized quantitative assay. In addition, neutralizing antibodies were measured using a commercial surrogate ELISA-assay. Interferon-gamma release was measured after stimulating blood-cells with SARS-CoV-2 specific peptides using a commercial assay to evaluate the cellular immune response.

**Results:**

We included 243 health-care workers who provided blood samples and questionnaires pre- and post- third vaccination. The median antibody level increased significantly after the third vaccination dose to 2663.1 BAU/ml vs. 101.4 BAU/ml (p < 0.001) before administration of the booster dose. This was also detected for neutralizing antibodies with a binding inhibition of 99.68% ± 0.36% vs. 69.06% ± 19.88% after the second dose (p < 0.001). 96.3% of the participants showed a detectable T-cell-response after the booster dose with a mean interferon-gamma level of 2207.07 mIU/ml ± 1905 mIU/ml.

**Conclusion:**

This study detected a BMI-dependent antibody increase after the third dose of BNT162b2 following different vaccination protocols. All participants showed a significant increase in their immune response. This, in combination with the low rate of post-vaccination-symptoms underlines the potential beneficial effect of a BNT162b2-booster dose.

## 1 Introduction

Following an initially flattened curve of COVID-19 infections, due to vaccination campaigns all over the world, there came a subsequent resurgence of COVID-19 infections worldwide (1). New variants of SARS-CoV-2 (2, 3) and decreasing immunity after vaccination over time (4, 5), have caused an increased rate of infection and hospitalization even in vaccinated individuals (1). A well-established approach to handle the so-called secondary vaccine failure, is the use of a booster dose – an additional vaccine dose after the initial round of immunization (1, 6). The effectiveness of a third dose of BioNTech/Pfizer vaccine was suggested by several studies (7–9). Therefore, after approval by the regulatory authorities in US (10) and in the European Union (11), several countries such as Israel, USA, UK and Germany, initiated a vaccination program for administration of a third dose in vulnerable groups at least 5 months after a complete initial course of vaccination (1, 12). The effect of this third dose on the immune response and the resulting protective effects have been analyzed in several registry and clinical studies (1, 9, 13, 14).

As the impact of different vaccines on the immune system varies, considering both cellular and humoral immunity is crucial. The detected levels of neutralizing antibodies have been shown to be higher after administration of the BioNTech/Pfizer vaccine BNT162b2 than those after receiving the AstraZeneca vaccine ChAdOx1, although this could not be shown for T-cell responses (15). These differences have led to discussion regarding potential benefit from a heterologous vaccination strategy (15–18).

We aimed to evaluate the humoral- and cellular immune response after a third booster-dose of BNT162b2. The booster dose was given following an initial administration of either two doses of BNT162b2 (Group 1), two doses of ChAdOx1 (Group 2) or cross-vaccination of BNT162b2+ChAdOx1 (Group 3) in a real-world setting analyzing one of the most important groups in this global pandemic – health care workers.

## 2 Methods

### 2.1 Study Design

In this study we evaluated the effect of administration of a third dose of BioNTech/Pfizer mRNA vaccine BNT162b2, within a longitudinal study in health care workers initiated in April 2020 (19). All employees of the Hospital Reinbek St. Adolf-Stift, a secondary care hospital located in Northern Germany, were eligible to participate in the study. The vaccination program for employees was established in December 2020. At the end of October 2021, the Robert-Koch-Institute recommended administration of a third dose in specific groups (20). Following this recommendation, beginning in November 2021 all employees who had received their second vaccination more than 6 months prior, were invited for a third booster-dose. In order to have a reference value for the determination of the booster-effect, blood samples were initially collected and analyzed before the administration of the third vaccine dose on November 13^th^ – 14^th^ 2021 (4).

To evaluate the efficiency of the third dose, all participants were asked to provide an additional blood sample and complete a questionnaire 4 weeks after the booster-vaccination (December 13^th^ – 14^th^ 2021).

### 2.2 Vaccination Protocol

The vaccination program for employees was established in December 2020. The vaccination was administered using either the mRNA vaccine of BioNTech/Pfizer or the vector-vaccine from AstraZeneca. This was necessary to ensure a timely vaccination for all employees. Both vaccines use the prime-boost protocol and the second dose of BNT162b2 was administered 42 days after the primary dose whereas the second dose of AstraZeneca was given 70 days later in accordance with the Robert-Koch-Institute. Due to the recommendations of the national regulatory board at the Robert-Koch-Institute, a heterogeneous vaccination was offered to all participants after a primary vaccination with a vector-based vaccine.

### 2.3 Anti-SARS-CoV-2-IgG Antibodies

The anti-SARS-CoV-2-IgG Antibody-titer was expressed in Binding Antibody Units per ml (BAU/ml) to stay in accordance with the WHO standard. Antibodies were determined by using a fully automated quantitative anti-SARS-CoV-2-assay (IgG) from Abbott (Chicago, USA). This is a chemiluminescent microparticle immunoassay (CMIA) which measures the IgG antibodies against the spike receptor-binding domain (RBD) of SARS-CoV-2. As recommended by the manufacturer, a value below 7.1 BAU/ml was determined to be negative whereas values ≥ 7.1 BAU/ml were determined to be positive.

### 2.4 Neutralizing Antibodies Against SARS-CoV-2

To evaluate the neutralizing anti-SARS-CoV-2 antibodies, all samples were additionally tested using the NeutraLISA™ SARS-CoV-2 Neutralization Antibody Detection KIT (Euroimmun, Lübeck, Germany). Results were presented in binding inhibition, where values ≥ 35% were considered positive. In contrast to the widely established neutralization assays using cell-cultures, this ELISA-based surrogate assay can be easily be performed in all laboratories and has showed a good correlation to the known cell-culture based assays (21). Even a selective testing against new variants is not possible in the used ELISA-based assay, in silico analyses according to the manufacturer’s instructions, suggest strongly that the CE marked test system used in the present study will not be negatively impaired by variants such as Omicron (22).

### 2.5 T-Cell-Response

In addition to the humoral immune response, the cellular immunity to SARS-CoV-2 was also assessed using the Quan-T-cell SARS-CoV-2 kit (Euroimmung, Lübeck, Germany) working as an Interferon-gamma release assay (IGRA). The analysis for this Quan-T-cell test was performed as previously reported (4). IFN-gamma concentration was expressed as mIU/ml. Values ≥ 200 mIU/ml were considered positive

### 2.6 Questionnaire

In the initial phase of this study in April 2020, participants completed a questionnaire regarding their medical history, including current medication and smoking habits. Before and after administration of the third dose of vaccine, all participants were asked to complete a questionnaire regarding their prior vaccinations, current smoking behaviors, and in addition after the third vaccination dose, possible side effects related to the booster dose. Specifically, participants were questioned about localized symptoms (local pain, lymphadenopathy) and systemic symptoms, such as fever or fatigue.

This study was conducted in accordance with the Declaration of Helsinki. All participants provided written and informed consent before inclusion. This study was prospectively registered at the German Clinical Trial Register (DRKS00021270) after approval by the Ethics Committee of the Medical Association Schleswig-Holstein.

### 2.7 Statistical Analysis

All variables are presented as means or medians with standard deviation or interquartile range. Categorical variables are shown as numbers with percentages. Statistical analysis was made using IBM SPSS Statistics Version 25 (IBM Co., Armonk, NY, USA). In addition, GraphPad Prism 9 was used for graphics.

Relationships between categorial variables were tested using the Chi-square test or the Fisher’s exact test, depending on the size of the groups. Inter-group differences were analyzed using Mann-Whitney-U test or Kruskal-Wallis-test reporting the mean with standard deviation. Pre- and post-third dose antibody and INF-gamma levels were compared using Wilcoxon sign rank test. A p-value < 0.05 was considered statistically significant.

To investigate the joint effect of age, sex, body mass index (BMI) and current smoking on the relative increase of antibody-level, a linear regression analysis was performed to take the antibody level before booster into account. The IgG-levels had a skewed distribution and was logarithmized for the regression analysis yielding an approximately normal distribution. As the dependent variable, the difference of the logarithmized IgG-levels after and before booster was used. The backward selection method was used to determine the final model.

## 3 Results

Before administration of the third vaccine-dose, 310 participants provided a blood specimen, of which 243 resubmitted a blood sample and completed questionnaire exactly 4 weeks after the booster dose (follow-up-rate 78.39%).

The participants included 59 (24.3%) male and 184 (75.7%) female participants with a mean age of 46.33 ± 11.44 years. The characteristics of the study cohort are described in **Table 1**, grouped according to their initial vaccination protocol.

**Table 1:** Characteristics of the study cohort (n = 243).

	All (n = 243)	Group 1 (n = 179)	Group 2 (n = 50)	Group 3 (n = 12)
**Age,** M ± SD [years]	46.33 ± 11.44	46.52 ± 10.99	43.94 ± 12.13	51.50 ± 13.62
**Sex,** n (%)				
** Male**	59 (24.3)	45 (25.1)	11 (22.0)	2 (16.7)
** Female**	184 (75.7)	134 (74.9)	39 (78.0)	10 (83.3)
**BMI,** M ± SD [kg/m^2^]	25.95 ± 5.02	26.00 ± 5.08	26.47 ± 5.09	23.57 ± 3.20
**Smoking,** n (%)	56 (23.0)	47 (26.3)	8 (16.0)	1 (8.3)
**Comorbidities,** n (%)				
** Cardiac**	44 (18.1)	33 (18.4)	8 (16.0)	3 (25.0)
** Pulmonary**	20 (8.2)	13 (7.3)	5 (10.0)	2 (16.7)
** Metabolic**	39 (16.0)	30 (16.8)	7 (14.0)	2 (16.7)
** Immunologic**	7 (2.9)	6 (3.4)	1 (2.0)	0 (0)
**Other**	50 (20.6)	39 (21.8)	8 (16.0)	3 (25.0)
**Reaction after booster,** n (%)	189 (77.8)	139 (77.7)	40 (80.0)	8 (66.7)
** Local pain**	147 (60.5)	109 (60.9)	31 (62.0)	5 (41.7)
** Headache**	49 (20.2)	32 (17.9)	13 (26.0)	4 (33.3)
** Fatigue**	91 (37.4)	61 (34.1)	26 (52.0)	3 (25.0)
** Fever**	30 (12.3)	21 (11.7)	9 (18.0)	0 (0)
** Limb pain**	47 (19.3)	34 (19.0)	10 (20.0)	3 (25.0)
** Lymphadenopathy**	12 (4.9)	11 (6.1)	0 (0)	1 (8.3)

M, mean; SD, standard deviation; BMI, body mass index. Two participants (1 male, 1 female) have had a different immunization protocol and are therefore not represented in one of the groups above.

All participants received a booster dose of BNT162b2, irrespective of their primary vaccination protocol. The participants included 179 (73.7%) individuals who had previously received two doses of BNT162b2 (Group 1), 50 (20.6%) individuals who had received heterologous vaccinations with BNT162b2+ChAdOx1 (Group 2), 12 (4.9%) individuals who were double vaccinated with ChAdOx1 (Group 3), and 2 (0.8%) individuals who had a natural SARS-CoV-2 infection followed by a single dose of BNT162b2.

### 3.1 Whole Study Cohort

#### 3.1.1 Anti SARS-CoV-2-IgG Binding Antibodies

**Figure 1:**
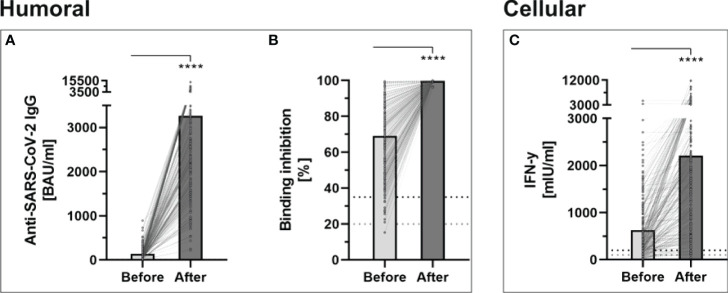
Determination of humoral- and cellular immunity against-SARS-CoV-2 before and four weeks after third booster vaccination with BNT162b2 (n = 243). **(A)** Anti-SARS-CoV-2-IgG binding antibodies were determined using a quantitative assay from Abbott. In keeping with the WHO-standard, data were expressed in Binding Antibody Units per ml (BAU/ml). Samples were marked seronegative below 7.1 BAU/ml whereas values above 7.1 BAU/ml were determined to be positive, as mentioned by the manufacturer. **(B)** Binding inhibition capability of Neutralizing anti-SARS-CoV-2 antibodies was determined using the NeutraLISA™ SARS-CoV-2 Neutralization Antibody Detection KIT from Euroimmun. According to the manufacturer, binding inhibition values above 35% were considered positive (horizontal black dotted line), whereas values between 20% and 35% were considered equivocal (horizontal gray dotted line). **(C)** Cellular immunity to SARS-CoV-2 was assessed by using an Interferon (IFN)-gamma release assay (IGRA) from Euroimmun (Quan-T-cell SARS-CoV-2 kit). Values > 200 mIU/ml were considered positive (horizontal black dotted line), whereas values between 100-200 mIU/ml were considered equivocal (horizontal gray dotted line). ****p < 0.0001 (Mann-Whitney U-test).

Prior to the booster dose, all participants still showed an antibody level above the manufacturer’s cutoff (> 7.1 BAU/ml). The median antibody level increased significantly after the third dose, when the whole study cohort was considered (2663.1, IQR 1700.7-4180.9 vs. 101.4, IQR 60.6-163.6; p < 0.001). All individuals showed increased antibody-levels with a median increase of 2539.55, IQR 1613.2-4002.5 (**Figure 1A**). The median relative increase was 25.9 (IQR 15.5-48.7).

#### 3.1.2 Neutralizing Antibodies

The administration of a third dose of BNT162b2 vaccine also caused a significant increase in neutralizing antibodies, throughout all study groups (99.68% ± 0.36% vs. 69.06% ± 19.88%; p < 0.001) (**Figure 1B**). No participants showed a binding inhibition capability below 96% following the booster-vaccination.

#### 3.1.3 T-Cell Response

Before the booster dose in November 2021, 73.4% of the participants still had a detectable T-cell response. This rate increased in the evaluation after the third dose to 96.3%, with a significantly higher mean INF-gamma level (2207.07 ± 1905.55 vs. 630.21 ± 650.53; p < 0.001) (**Figure 1C**).

### 3.2 Vaccination-Strategy Specific Cohorts

#### 3.2.1 Anti SARS-CoV-2 Binding Antibodies

**Figure 2:**
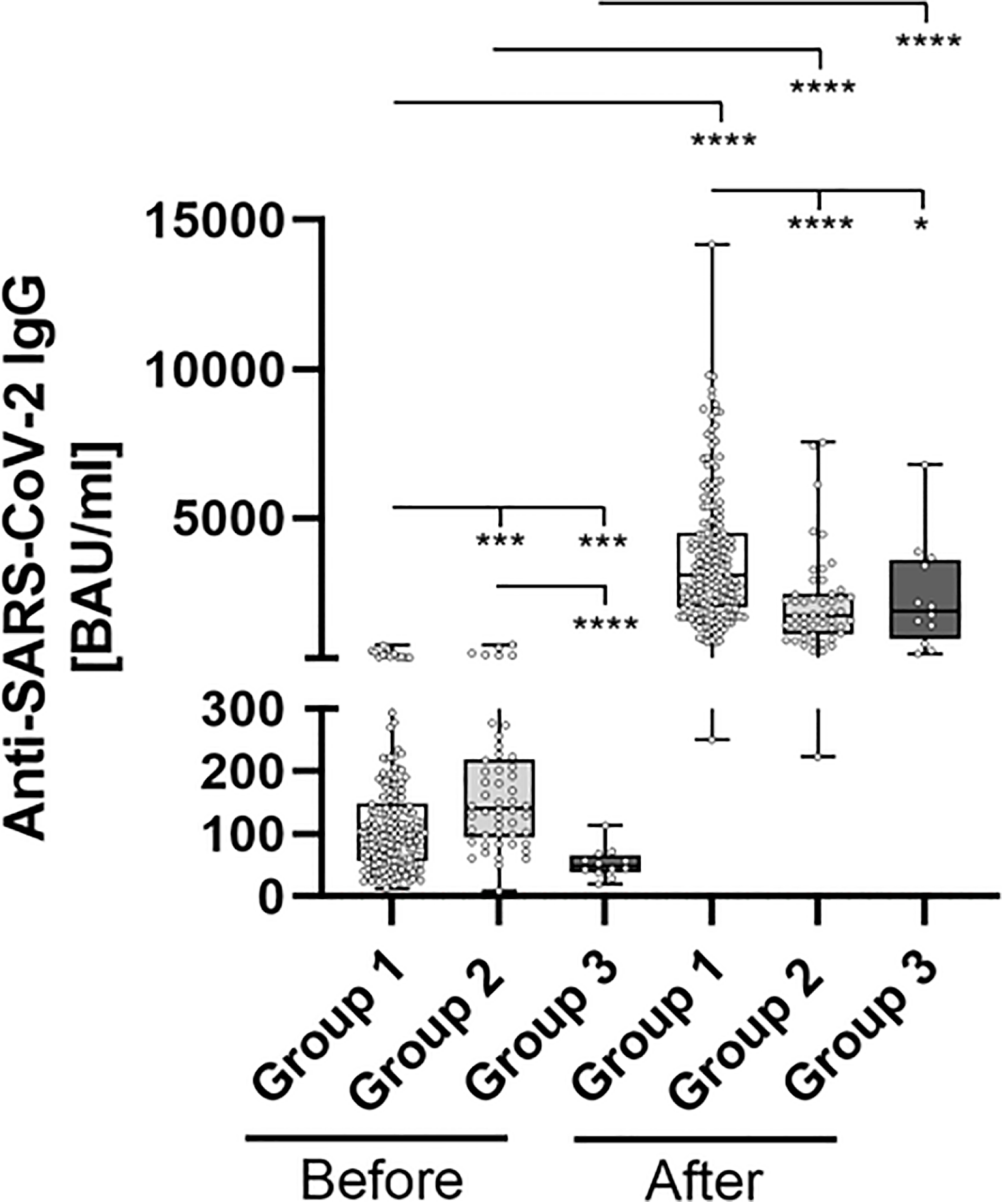
Comparative determination of anti SARS-CoV-2 IgG binding antibodies before and after third booster vaccination with BNT162b2. To evaluate differences between vaccination-strategies, participants were grouped into 3 cohorts: Group 1: three vaccine-doses of BNT162b2; Group 2: initially two vaccine-doses of ChAdOx1 and BNT162b2 booster-dosage; Group 3: heterologous vaccination-protocol (ChAdOx1+ BNT162b2) and BNT162b2 booster-dosage. Anti-SARS-CoV-2-IgG binding antibodies were determined before and 4 weeks after third booster-dosage using a quantitative assay from Abbott. In keeping with the WHO-standard, data were expressed in Binding Antibody Units per ml (BAU/ml). Samples were marked seronegative below 7.1 BAU/ml whereas values above or equal to 7.1 BAU/ml were determined to be positive, as mentioned by the manufacturer. *p < 0.05; ***p < 0.001; ****p < 0.0001 (Mann-Whitney U-test).

Anti-SARS-CoV-2 binding antibody levels differed significantly between the subgroups (representing different prior vaccination protocols), prior to booster-administration. In brief, pre-boost antibody levels were highest in Group 2 (183.1 BAU/ml), followed by Group 1 (124.9 BAU/ml) and Group 3 (52.75 BAU/ml). The administration of a third BNT162b2 dose led to a highly significantly increased antibody-level, across all analyzed subgroups. Inductive effects were highest within Group 3 participants (mean 2358 vs. 52.75 (4,370%), p = 0.002), followed by Group 1 (mean 3661 vs. 124.9 (2,831%), p < 0.001) and Group 2 (mean 2122 vs. 183.1 (1,058%), p < 0.001) participants (**Figure 2** and **Table 2**).

**Table 2:** Values in accordance to the immunization protocol.

	All			Group 1			Group 2			Group 3		
	IgG [BAU/ml]	INF-y [mlU/ml]	NAK [%]	IgG [BAU/ml]	INF-y [mlU/ml]	NAK [%]	IgG [BAU/ml]	INF-y [mlU/ml]	NAK [%]	IgG [BAU/ml]	INF-y [mlU/ml]	NAK [%]
**All**	3266.26 ± 2194.17	2207.07 ± 1905.55	99.68 ± 0.36	3660.88 ± 2250.13	2013.70 ± 1780.13	99.71 ± 0.28	2357.80 ± 1815.63	3090.58 ± 2671.98	99.70 ± 0.18	2122.32 ± 1572.44	2424.93 ± 1520.45	99.59 ± 0.57
**Sex**												
**Male**	3397.52 ± 2675.29	2010.27 ± 1526.05	99.65 ± 0.52	4064.25 ± 2716.71	2130.64 ± 1527.94	99.74 ± 0.16	1032.35 ± 707.32	771.40 ± 274.99	99.51 ± 0.28	1232.95 ± 707.32	1428.37 ± 1133.33	99.31 ± 1.13
**Female**	3224.17 ± 2022.59	2270.53 ± 2012.33	99.69 ± 0.29	3525.42 ± 2064.21	1974.13 ± 1861.38	99.70 ± 0.31	2622.89 ± 1872.16	3554.42 ± 2698.76	99.73 ± 0.14	2373.17 ± 1662.26	2706.01 ± 1508.11	99.66 ± 0.23
**Smoking**												
**Yes**	3419.37 ± 2328.14	1649.21 ± 1347.08	99.71 ± 0.22	3737.61 ± 2390.63	1545.40 ± 1338.35	99.74 ± 0.12	2156.10	3934.84	99.61	1707.60 ± 880.06	1973.41 ± 1244.33	99.50 ± 0.47
**No**	3220.41 ± 2156.84	2375.03 ± 2016.98	99.68 ± 0.39	3633.56 ± 2206.79	2181.71 ± 1890.24	99.70 ± 0.32	2376.14 ± 1903.08	3013.83 ± 2788.49	99.70 ± 0.18	2201.32 ± 1668.18	2510.93 ± 1565.61	99.60 ± 0.59
**Obesity**												
**Yes**	3772.66 ± 2586.61	1805.01 ± 1499.21	99.72 ± 0.10	4159.60 ± 2769.73	1501.28 ± 1307.93	99.72 ± 0.10	N/A	N/A	N/A	2760.68 ± 1737.00	2599.36 ± 1722.37	99.73 ± 0.09
**No**	3144.83 ± 2078.43	2303.98 ± 1982.24	99.67 ± 0.39	3543.94 ± 2104.28	2134.69 ± 1857.52	99.71 ± 0.31	2357.80 ± 1815.63	3090.58 ± 2671.98	99.70 ± 0.18	1898.04 ± 1469.99	2363.64 ± 1463.86	99.53 ± 0.66
**Symptoms after vaccination**												
**Yes**	3397.22 ± 2277.30	2394.65 ± 1970.57	99.67 ± 0.39	3787.36 ± 2347.85	2203.38 ± 1891.74	99.70 ± 0.31	2768.40 ± 1962.11	3008.23 ± 1952.78	99.70 ± 0.18	2237.24 ± 1642.77	2622.83 ± 1601.94	99.57 ± 0.62
**No**	2807.92 ± 1819.79	1538.16 ± 1485.19	99.71 ± 0.18	3221.36 ± 1829.71	1337.65 ± 1078.61	99.73 ± 0.12	1536.60 ± 1324.94	3255.29 ± 4150.42	99.70 ± 0.18	1662.66 ± 1214.08	1633.30 ± 771.86	99.66 ± 0.33

IgG, Immunoglobulin G; INF-y, interferon gamma; NAK, neutralizing antibodies; N/A, not available. Values are reported as mean ± standard deviation except where otherwise specified.

#### 3.2.2 Neutralizing Antibodies

**Figure 3:**
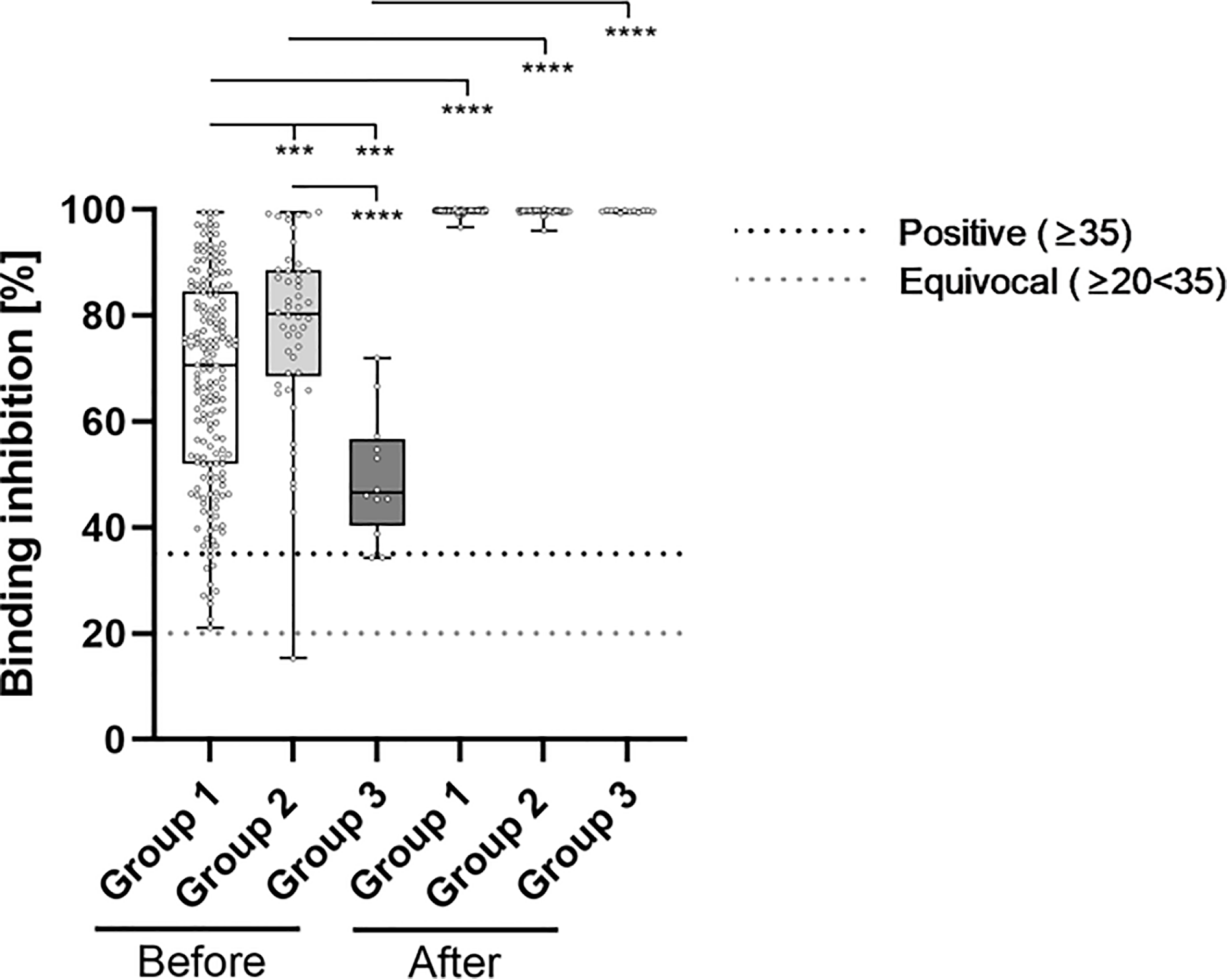
Comparative determination of neutralizing antibody binding-inhibition-capability before and after third booster vaccination with BNT162b2. To evaluate differences between vaccination-strategies, participants were grouped into 3 cohorts: Group 1: three vaccine-doses of BNT162b2; Group 2: initially two vaccine-doses of ChAdOx1 and BNT162b2 booster-dosage; Group 3: heterologous vaccination-protocol (ChAdOx1+ BNT162b2) and BNT162b2 booster-dosage. Binding inhibition capability of neutralizing anti-SARS-CoV-2 antibodies was determined using the NeutraLISA™ SARS-CoV-2 Neutralization Antibody Detection KIT from Euroimmun. According to the manufacturer, binding inhibition values above or equal to 35% were considered positive (horizontal black dotted line), whereas values between 20% and 35% were considered equivocal (horizontal gray dotted line). ***p < 0.001; ****p < 0.0001 (Mann-Whitney U-test).

Neutralizing antibody levels also differed significantly between the groups prior to booster-administration (**Figure 3** and **Table 2**). Specifically, individuals within Group 3 showed the lowest percentage binding inhibition (49.55%), followed by Group 1 (67.66%) and Group 2 (77.57%) participants. The administration of a third BNT162b2 dose caused a significant induction of binding antibody capability within all three groups, whereby the strongest effect was detected for Group 3 (99.70% vs. 49.55% (induction: 101.21%)), followed by Group 1 (99.71% vs. 67.66% (induction: 47.37%)) and Group 2 (99.59% vs. 77.57% (induction: 28.39%)) participants. Post-booster binding inhibition capabilities did not differ significantly between the different groups.

#### 3.3.3 T-Cell Response

**Figure 4:**
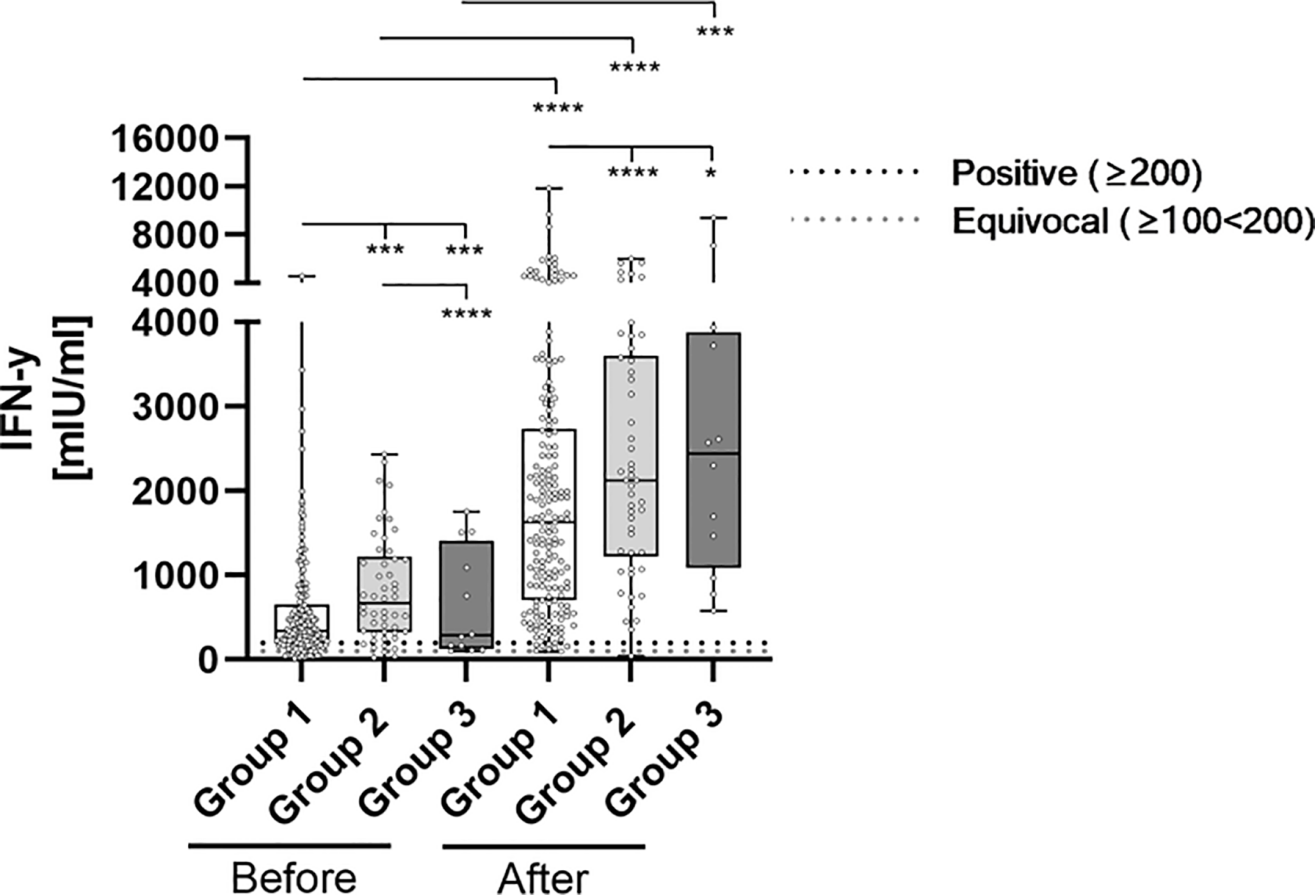
Comparative determination of SARS-CoV-2 specific t-cell response before and after third booster vaccination with BNT162b2. To evaluate differences between vaccination-strategies, participants were grouped into 3 cohorts: Group 1: three vaccine-doses of BNT162b2; Group 2: initially two vaccine-doses of ChAdOx1 and BNT162b2 booster-dosage; Group 3: heterologous vaccination-protocol (ChAdOx1+ BNT162b2) and BNT162b2 booster-dosage. Cellular immunity to SARS-CoV-2 was assessed by using an Interferon (IFN)-gamma release assay (IGRA) from Euroimmun (Quan-T-cell SARS-CoV-2 kit). Values ≥ 200 mIU/ml were considered positive (horizontal black dotted line), whereas values between 100-200 mIU/ml were considered equivocal (horizontal gray dotted line). *p < 0.05; ***p < 0.001; ****p < 0.0001 (Mann-Whitney U-test).

Prior to the administration of a booster BNT162b2 dosage, SARS-CoV-2 specific IFN-gamma release of stimulated blood-cells significantly differed between individuals of each considered group (**Figure 4**; **Table 2**). Briefly, Group 1 participants showed the lowest IFN-gamma mean-value (560.4 mIU/ml), followed by individuals belonging to Group 3 (654.7 mIU/ml) and Group 2 (828.7 mIU/ml). SARS-CoV-2 specific T-cell response significantly increased in boostered individuals within all groups. The strongest percentage inductive effects were observed for Group 3 individuals [3091 vs. 654.7 (372.12%)], followed by participants belonging to Group 1 [2014 vs. 560.4 (259.39%)] and Group 2 [2425 vs. 828.7 (192.63%)].

### 3.4 Factors Impacting the Immune Response

In the linear regression analysis, we identified the BMI as a significant predictor for the antibody-level increase. The increase was also dependent on the antibody-level before booster. Neither sex, age or smoking had a significant effect. The estimated effect of a previous COVID-19 infection is negative, however since very few individuals (n = 2) had a prior infection, no conclusion can be drawn from this result. **Table 3** gives the result of the full regression model. The fit of the model was very good with an R^2^ value of 0.43. The regression coefficients remained virtually unchanged when only the significant variables, BMI and antibody-level before booster, remained in the final model (**Table 3**).

**Table 3:** Results of the linear regression analysis.

Dependent variable difference of the logarithmized IgG-levels after and before booster				
Independent Variables	Full model		Final model	
	Estimate	p	Estimate	p
Intercept)	5.518	<.0001	5.57	<0.0001
Log(IgG) (before Booster)	-0.620	<.0001	-0.64	<0.0001
Age (in years)	0.002	0.58		
BMI	0.023	0.005	0.024	0.003
Smoking	-0.101	0.30		
Sex (male vs. female)	-0.049	0.60		
Previous COVID	-0.213	0.51		
Model R^2^	0.43		0.43	

We compared the immune status before the third dose showing that obese participants have a lower mean anti-SARS-CoV-2 binding antibody level in comparison with the non-obese participants (111.28 BAU/ml vs. 143.96 BAU/ml; p = 0.082). This was also seen for the neutralizing antibodies (64.19% vs. 70.23%; p = 0.071).

From the regression model, the estimated antibody-level showed both an absolute and relative increase given the BMI and the antibody-level before booster. **Table 4** provides the estimates for some selected values. For example, an individual with an antibody-level of 100 prior to booster and BMI of 25, has an estimated value of 2552 after the booster, which is an absolute increase of 2452 and 25.5-fold relative increase.

**Table 4:** Estimates for different selected values.

BMI	antibody-level before	Estimated values from regression model		
		antibody-level after	Ratio after:before	Difference after-before
20	50	1748.6	35.0	1698.6
	100	2275.5	22.8	2175.5
	200	2961.2	14.8	2761.2
	400	3853.6	9.6	3453.6
25	50	1961.7	39.2	1911.7
	100	2552.8	25.5	2452.8
	200	3322.1	16.6	3122.1
	400	4323.2	10.8	3923.2
30	50	2200.8	44.0	2150.8
	100	2864.0	28.6	2764.0
	200	3727.0	18.6	3527.0
	400	4850.1	12.1	4450.1

BMI, Body mass index.

### 3.5 Side Effects of Third Dose

The majority of the study cohort reported some minor side-effect following the booster dose (**Table 1**). The occurrence of side-effects did not differ between all three groups (p = 0.666). Headache was more often reported in participants after two doses of ChAdOx1 (33.3%) in comparison to the other groups (p = 0.329). All other reported side effects occurred less often in that group, however, this group only includes 12 participants. 54 participants (22.2%) reported no systemic or local reaction after administration of the third dose. These participants without side-effects have had a mean age of 49.28 years whereas the mean age in the group with side-effects was 45.49 years (p = 0.051).

## 4 Discussion

We present real-life data showing the impact of administration of a third dose of COVID-19 vaccination using BNT162b2 mRNA vaccine, following different vaccination protocols in a well-defined group of health care employees. Our data reveal a significantly increased cellular and humoral immune response to this additional vaccination dose.

Our data show that both humoral- and cellular immunity appear to be most persistent after double ChAdOx1 vaccination compared to the other vaccination strategies included in the study. In contrast, Dulovic et al. in their study showed that the persistence of neutralizing antibodies is shortest in individuals who have received the homologous ChAdOx1 vaccine. Contrarily, the administration of a heterologous- or a homologous mRNA strategy led to a stronger persistence. These discrepancies can be explained by the fact that Dulovic et al. only examined persistence over a maximum period of 65 days following administration of the second vaccine dose (23). In our study, we considered a much longer time period of 6 months.

Our study shows the highest increase in INF-gamma release, binding-antibody expression, as well as neutralizing antibody capability for participants initially vaccinated two times with ChAdOx1. However, these participants showed the weakest humoral immune response before administration of the booster dose. A regression analysis detected a high BMI to be associated with an increased immune response.

Several studies have analyzed the effect of a third dose on the immune response and on the occurrence of infections, and showed an overall beneficial effect of the third dose, without relevant adverse events (8, 24–29).

Atmar et al. analyzed in their phase 1-2 open-label clinical trial the efficiency of homologous and heterologous protocols for the third dose, and found a stronger increase in immunity against SARS-CoV-2 following a heterologous third dose (30). This is also shown in the data presented here.

In addition to the determined overall binding antibody titers, the neutralizing antibodies play a main role in the humoral immune response and are correlated with protection against severe COVID-19 outcome (31).

Using the commercial ELISA-based NeutraLISA assay from Euroimmun, our data show a substantial increase in neutralizing antibody capability in all participants after booster-administration. This is in line with the data presented from Atmar et al. (30). Data from US and Israel underline the protective effect of a third dose of BNT162b2 against a severe COVID-19-course (8, 32, 33).

### 4.1 Factors Causing a Reduced Immune Response

Multiple studies have tried, and are still attempting, to evaluate possible factors associated with a reduced immune response or with a complete non-response to anti-SARS-CoV-2-vaccination.

Within the study cohort presented here, smoking, increased age and BMI have previously been shown to have a negative impact in studies involving two doses of BNT162b2 (34) or 9 months after vaccination, respectively (4). This could not be reproduced in the results presented here (**Table 3**). The impact of BMI on the vaccination induced immune response is described heterogeneously after several vaccines. Previous data from vaccination protocols, such as hepatitis B vaccine, range from no impact (35) to a reduced immune response in obese participants (36). Our data presented here showed a positive correlation of a higher BMI with a stronger increase of the antibody titer after the third dose within a short period of time. This may be due to a lower antibody titer before administration of the third dose as reported previously (4). Soffer et al. also found a positive correlation after COVID-19 infection for antibody titers and BMI, whereas Frasca et al. found the opposite correlation (37). The detected effect may also be due to the short period of time after vaccination. Sheridan et al. showed in their study on the immune response of obese participants to influenza vaccination a higher initial antibody level, which decline within 12 months after vaccination (38). In contrast to this, Pellini et al. found a lower antibody level immideatly after vaccination in obese participants (39) showing that the effect of BMI on the immune response remains still unclear.

Other factors potentially influencing the immune response after a third dose of BNT162b2 are immunosuppression, for instance in patients after solid-organ transplantation (24).

In our cohort, we could not detect any negative impact of older age, although elderly participants are relatively underrepresented in this trial. Data published by Eliakim-Raz et al. also described no correlation to higher age in a group older than 60 years (25).

These findings can aid in the development of a safe vaccination protocol, especially for patients with a potentially higher risk, for example because of a high BMI. It also provides real world data following different vaccination protocols, and helps to develop benchmarks for a potential immune response.

Further studies are required to provide more accurate information on risk factors associated with reduced immunity to SARS-CoV-2.

### 4.2 Limitation

Despite the fact this study benefits from its real-life data in an important group of health-care workers, and a relatively large number of participants, it comes with some limitations.

This study is limited by its single-center design. The inclusion of health-care workers led to the overrepresentation of women and younger people, whereas groups with a higher risk for low immune response to vaccination, such as elderly and participants with an immunomodulatory treatment, are underrepresented. To ensure a good comparability of the samples, we performed the follow-up within two days. This led to a lost-to-follow-up proportion of 21.6% of the previously screened participants, due to holidays or different working schedules. Unfortunately, the number of individuals in each group of vaccination protocols were unequally distributed, as there was no individual choice regarding the vaccine received. This led to a very small number of participants in Group 3, limiting the reported strong immune response. As this was designed as a serological study, the survey of side-effects was not in the main focus and is limited due to the retrospective evaluation. Additionally, this study is limited by the use of an ELISA-based surrogate neutralization test. Even this showed a good correlation to the gold standard of a cell-culture based neutralization assay, the testing against new variants is not possible. As this study aims to report the short-term-outcomes of the third dose within the COVID-19-vaccination protocol, the long-term effect, especially for debatable factors as the BMI, are missing.

## 5 Conclusion

This study showed a BMI-dependent increase in antibody titers after the third dose of BNT162b2 following different vaccination protocols, although all participants were noted to have a significant increase in their immune response. In this study, participants after two doses of ChAdOx1 who received BNT162b2 as a third dose showed the strongest immune response. This effect, in combination with mild-to-none post-vaccination symptoms, underlines the potential beneficial effect of a BNT162b2-booster dose following 2 doses of anti-SARS-CoV-2-vaccine. Further studies are needed to evaluate thresholds for the immune response – both humoral and cellular – and to detect the longevity of the booster-induced immunity.

## Data Availability Statement

The raw data supporting the conclusions of this article will be made available by the authors on reasonable request, without undue reservation.

## Ethics Statement

The studies involving human participants were reviewed and approved by Ethics Committee of the Medical Association Schleswig-Holstein, Germany. The participants provided their written informed consent to participate in this study.

## Author Contributions

JH and BF conducted the research and wrote the manuscript. These authors contributed equally to this research. HB supervised the statistical analysis. A-KB, HH, and SYG reviewed the manuscript. TS initiated the research and supervised the study. TS and CK reviewed the final draft of this article and provided logistic support. All authors have critically reviewed and approved the final draft and are responsible for the content of the manuscript.

## Conflict of Interest

The authors declare that the research was conducted in the absence of any commercial or financial relationships that could be construed as a potential conflict of interest.

## Publisher’s Note

All claims expressed in this article are solely those of the authors and do not necessarily represent those of their affiliated organizations, or those of the publisher, the editors and the reviewers. Any product that may be evaluated in this article, or claim that may be made by its manufacturer, is not guaranteed or endorsed by the publisher.
